# Supplementary material for: Nanoscale architecture of a VAP-A-OSBP tethering complex at membrane contact sites
Source: Nat Commun. 2021 Jun 8;12:3459. doi: 10.1038/s41467-021-23799-1 (PMC8187361; doi:10.1038/s41467-021-23799-1)
Supplement: Supplementary file 1 — Supplementary information [file 41467_2021_23799_MOESM1_ESM.pdf]

E. de la Mora et al.,

Nanoscale architecture of a VAP-A-OSBP tethering complex at membrane contact site

KEY RESOURCES TABLE

Supplementary Table 1

Supplementary Table 2

Supplementary Figures Legends

Supplementary Figure 1-5

Supplementary table 1 Data collection and image processing table

	**N-PH-FFAT**	**VAP-A**
Magnification	80 000	80 000
Voltage (kV)	300	300
Electron exposure (e^{-}/Å^2)	~145	~145
Defocus range (µm)	-2.0 to -4.8	-2.0 to -4.8
Energy filter slit width (eV)	20	20
Tilt range (min/max, step)	-60°/60°, 3°	-60°/60°, 3°
Pixel size (Å)	1.7	1.7
Tomograms (used/acquired)	50/90	30/90
Symmetry	c2	c1
Final/total subvolumes	15597	6948
Map resolution (FSC = 0.143)	9.8Å	19.6Å
EMDB-ID	11376	11402

Supplementary Table 2

KEY RESOURCES TABLE

REAGENT or RESOURCE	SOURCE	IDENTIFIER
**Bacterial and Virus Strains**		
C41 (DE3) Escherichia coli	Institut Curie ressources	N/A
BL21 (DE3) Escherichia coli	IPMC ressources	N/A
**Chemicals, Peptides, and Recombinant Proteins**		
DDM, N Dodecyl (β)- D Maltoside	Anatrace	Cat#D310 ;<br>CAS:69227-93-6
Anapoe-X-100 (Triton X100)	Anatrace	Cat#APX100 ;<br>CAS:9002-93-1
Egg phosphatidylcholine	Avanti Polar Lipids	Cat#840051C ;<br>CAS:97281-44-2
Brain phosphatidylserine	Avanti Polar Lipds	Cat#840032C;<br>CAS:383907-32-2
Rhodamine DHPE	Molecular Probes	Cat#L1392
C24:1 Galactosyl(ß) Ceramide	Avanti Polar Lipds	Cat#860546P;<br>CAS:17283-91-9
Brain PI4P	Avanti Polar Lipids	Cat#840045X;<br>CAS:475995-51-8
Texas Red DHPE<br>1,2-dihexadecanoyl-sn-glycero-3-phosphoethanolamine,<br>triethylammonium salt (Tx-DHPE)	Thermofisher Scientific	Cat#T1395MP
Bio-Beads SM2 Adsorbents	Bio-Rad	Cat#152-3920;<br>CAS:9003-70-7
Maleimide Alexa Fluor –488 C5	Thermofisher Scientific	Cat#A1025
Maleimide Alexa Fluor –568	Thermofisher Scientific	Cat#A20341
**Deposited Data**		

Raw and analyzed data	This paper	EMD-11455, EMD-11427, EMD-11402, EMD-11376, EMD-11399
**Oligonucleotides**		
VapA_FL_LIC_For: TACTTCCAATCCAATGCAATGGCGAAGCACGAGCAG		
VapA_FL_LIC_Rev: TTATCCACTTCCAATGTTATTACAAGATGAATTTCCCTAG		
**F.GST-OSBP-V199 :** GGAGATATACATGGGATCCGTCTCACAAACTGACAAG		
**F.GST-OSBP-V199C :** GGAGATATACATGGGATCCTGCTCACAAACTGACAAG		
**R.GST-OSBP1-G324 :** GAGATATACACCCGGGTCATCA**TCC**TCGGAAGGCCCTCT		
**R.GST-OSBP1-G324C :** GAGATATACACCCGGGTCATCA**ACA**TCGGAAGGCCCTCT		
**Recombinant DNA**		
pET16b. 6His.StrepII.TEV.VAPA (8-249)	This study	N/A
pET16b. StrepII.TEV.OSBP N-PH-FFAT (1-408)	[1]	N/A
OSBP		N/A
NBD-FAPP1 PH domain	[2]	N/A
pGEX-OSBP(198-324), N-ter or C-ter mutants	This study	N/A
**Software and Algorithms**	Mathworks	https://fr.mathworks.com

MotionCor2	[3]	https://emcore.ucsf.edu/ucsf-motioncor2
ImageJ	[4]	https://imagej.nih.gov/ij/
SerialEM	[5]	http://bio3d.colorado.edu/SerialEM/
Matlab R2019a	Mathworks	https://fr.mathworks.com
CTFPlotter and CTFphaseflip	[6]	https://bio3d.colorado.edu/imod/doc/man/ctfplotter.html
Dynamo	[7,8]	https://wiki.dynamo.biozentrum.unibas.ch/w/index.php/Main_Page
IMOD	[9]	http://bio3d.colorado.edu/imod/
UCSF Chimera	[10]	https://www.cgl.ucsf.edu/chimera/
Robetta	[11–13]	http://robetta.bakerlab.org
Phyres2	[14]	http://www.sbg.bio.ic.ac.uk/phyre2/html/page.cgi?id=index
PSIpred	[15]	http://bioinf.cs.ucl.ac.uk/psipred/
Pymol	[16]	https://pymol.org
Gromacs 2019.4	[17]	http://www.gromacs.org/

**Other**		
Peptidol:HS-PEG capped gold nanoparticles	[18]	N/A
Lacey formvar/carbon 300 mesh copper grids	Ted Pella (USA)	Cat#01883-F
IBA superflow sepharose		
Kit for protein concentration measurements		
Superdex 200 (GE healthcare)		

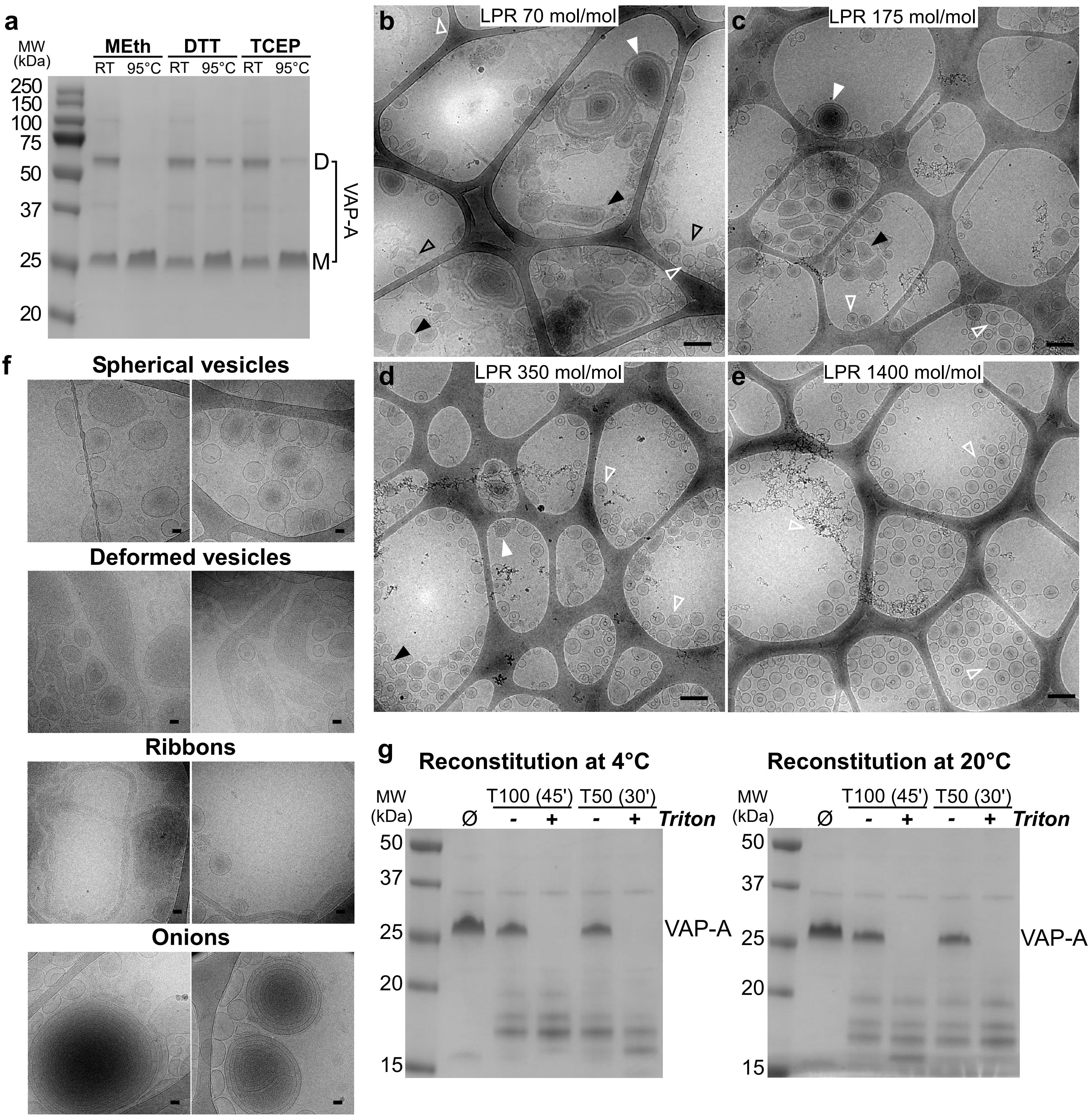

Supplementary figure 1

Supplementary Figure 1. Biochemical analysis of VAP-A and Cryo-EM images of reconstitution of VAP-A at different lipid/protein molar ratio.

(a) Purified full length of VAP-A in 12 % SDS PAGE gel in the presence of reducing agents at room temperature or after 5 min incubation at 95°C.The two bands were consistent with dimers and monomers of VAP-A.

(b) Reconstitution of VAP-A at lipid to protein ratio (LPR) 70, (c) 175, (d) 350, (e) 1400 mol/mol. Onions (full white arrow), deformed vesicles (full black arrow), ribbons (open black arrow), spherical liposomes (empty white arrow). Bars = 250 nm.

(f) Different types of reconstituted membranes containing of VAP-A at LPR 70 and 175 mol/mol, spherical and slightly deformed vesicles, deformed vesicles, ribbons and onions. Bars: 50 nm.

(g) Orientation of VAP-A in proteoliposomes in contact with N-PH-FFAT or OSBP. Orientation of VAP-A in proteoliposomes reconstituted at RT and at 4°C at LPR 70 mol/mol, as shown Figure 1e and figure 3c, respectively. Proteoliposomes were not treated (lane ϕ), digested at the indicated time incubation with trypsin at a VAP-A/Trypsin 100 or 50 mol/mol before or after (lanes T100) solubilization with Triton X100 (Triton X100/lipid ratio 2.5 w:w). VAP-A was oriented 40/60 out/in on average after reconstitution at RT and 50/50 after reconstitution at 4°C in small liposomes.

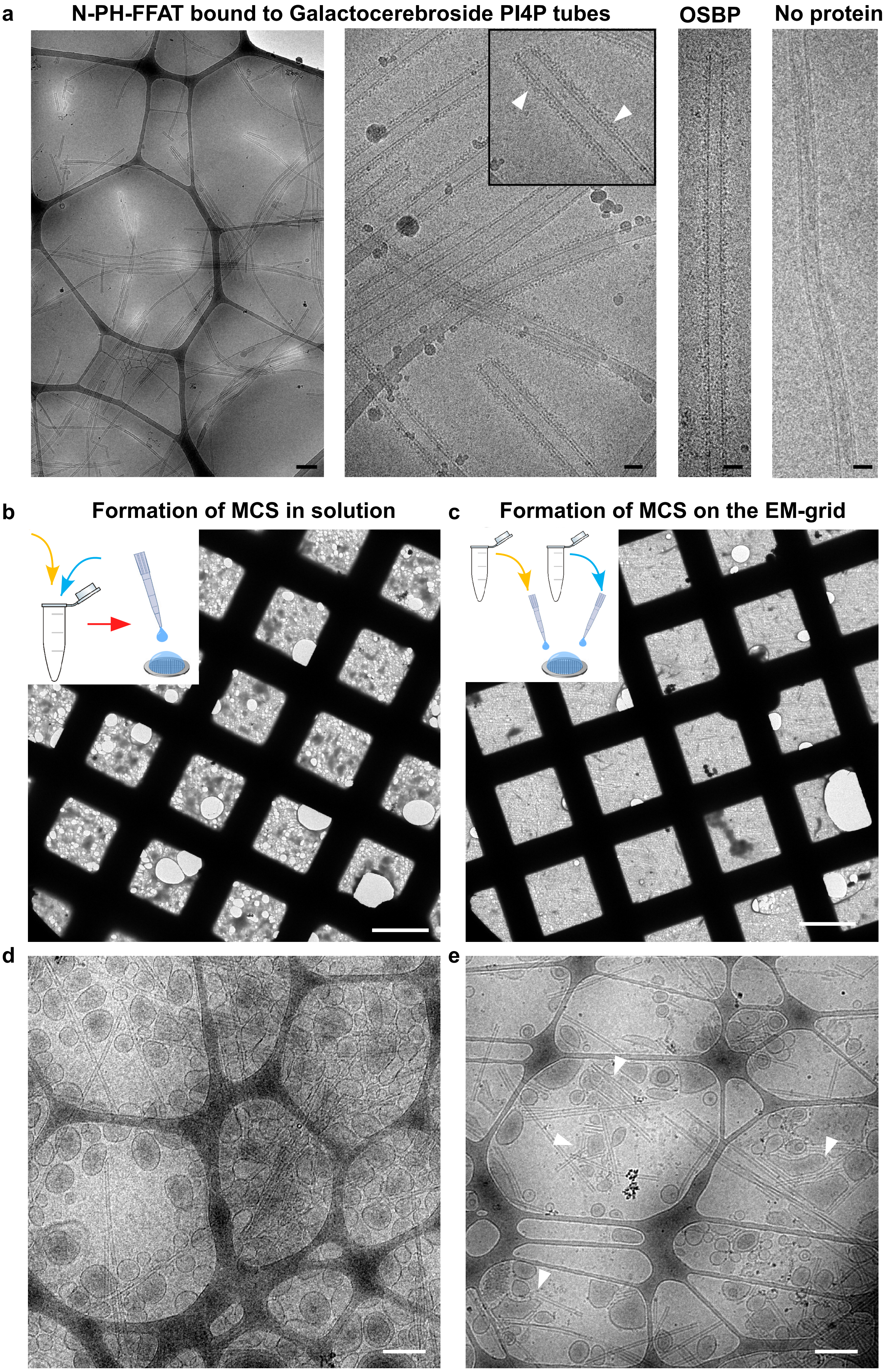

Supplementary figure 2

Supplementary Figure 2. Formation of in vitro membrane contact site.

(a) N-PH-FFAT or OSBP bound to galactocerebroside tubes doped with PI4P. Galactocerebroside tubes Galcer/EPC/PS/Pi4P (80/10/5/5) were mixed with N-PH-FFAT at 80 lipid/protein mol/mol. Tubes are covered with proteins that point to 6 nm from the membrane (white arrows Inset). Bars: 250 nm (left figure), 25 nm.

(b-e). Formation of membrane contact site for cryo-EM on cryo-EM grids. (b, d) The mixture of VAP-A proteoliposomes and N-PH-FFAT-tubes in an eppendorf before freezing lead to a massive aggregation of material and thick ice. (c, e) Sequential addition of VAP-A proteoliposomes and N PH-FFAT tubes on cryo-EM grids lead to the formation of membrane contact site (white arrow heads) suitable for cryo-EM and cryo-ET. Bars: 50 µm in (b, c) and 250 nm in (d, e).

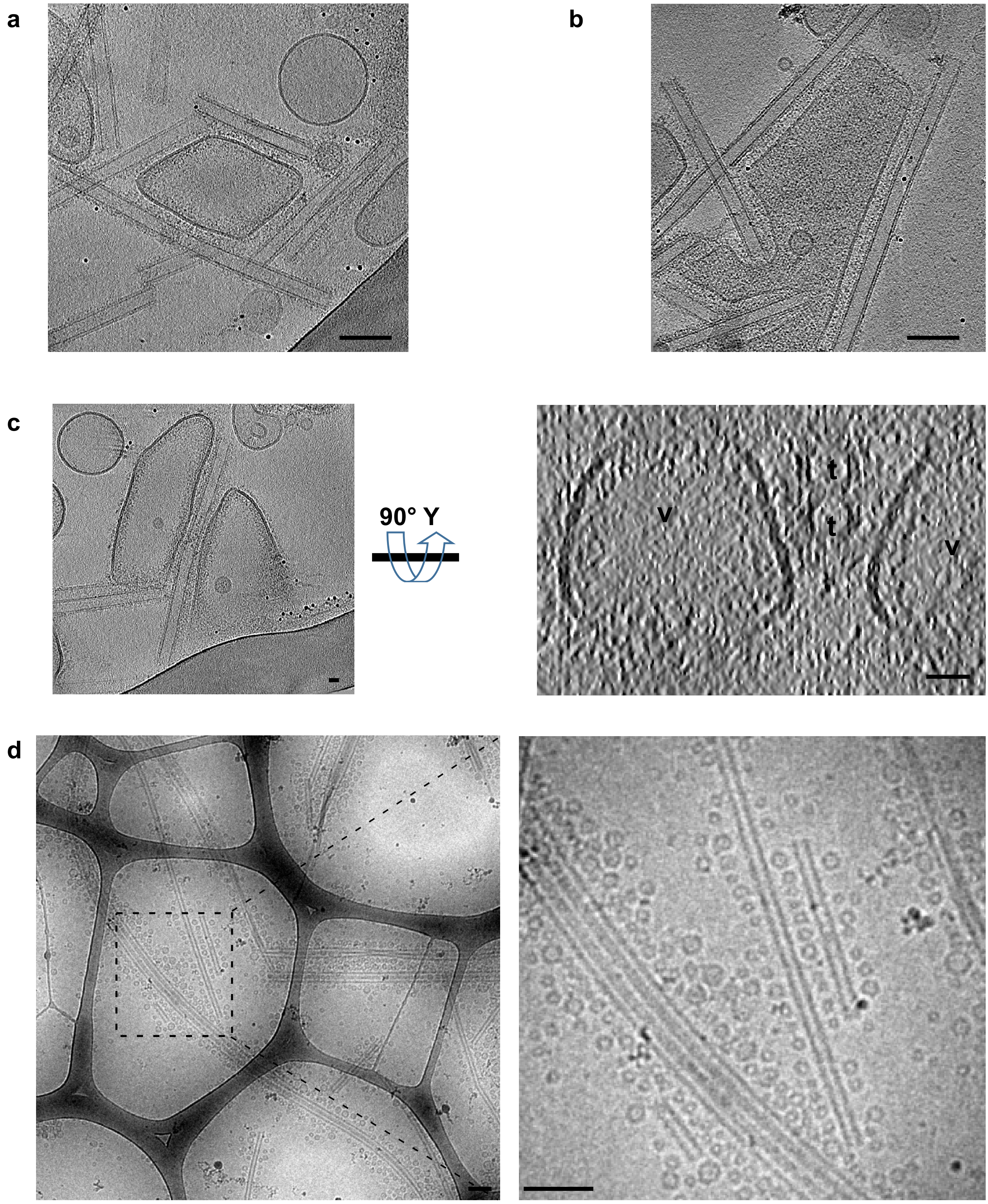

Supplementary Figure 3

Supplementary Figure 3. Membrane remodeling during formation of membrane contact sites (MCS).

(a, b) Tomographic slices of two tilt series of VAP-A vesicles remodeled in contact with 4 tubes. In B, the large vesicle went around the tube but did not wrapped around. Bars: 100 nm (c) Tomographic slice of a tilt series in XY and YZ plans. In YZ plan, VAP-A vesicles (v) are flattened in contact to two close tubes (t). Bars: 25 nm (d) Low magnification and inset of MCS made with small vesicles of VAP-A reconstituted at LPR 175 mol/mol and N-PH-FFAT tubes. Bars: 100 nm.

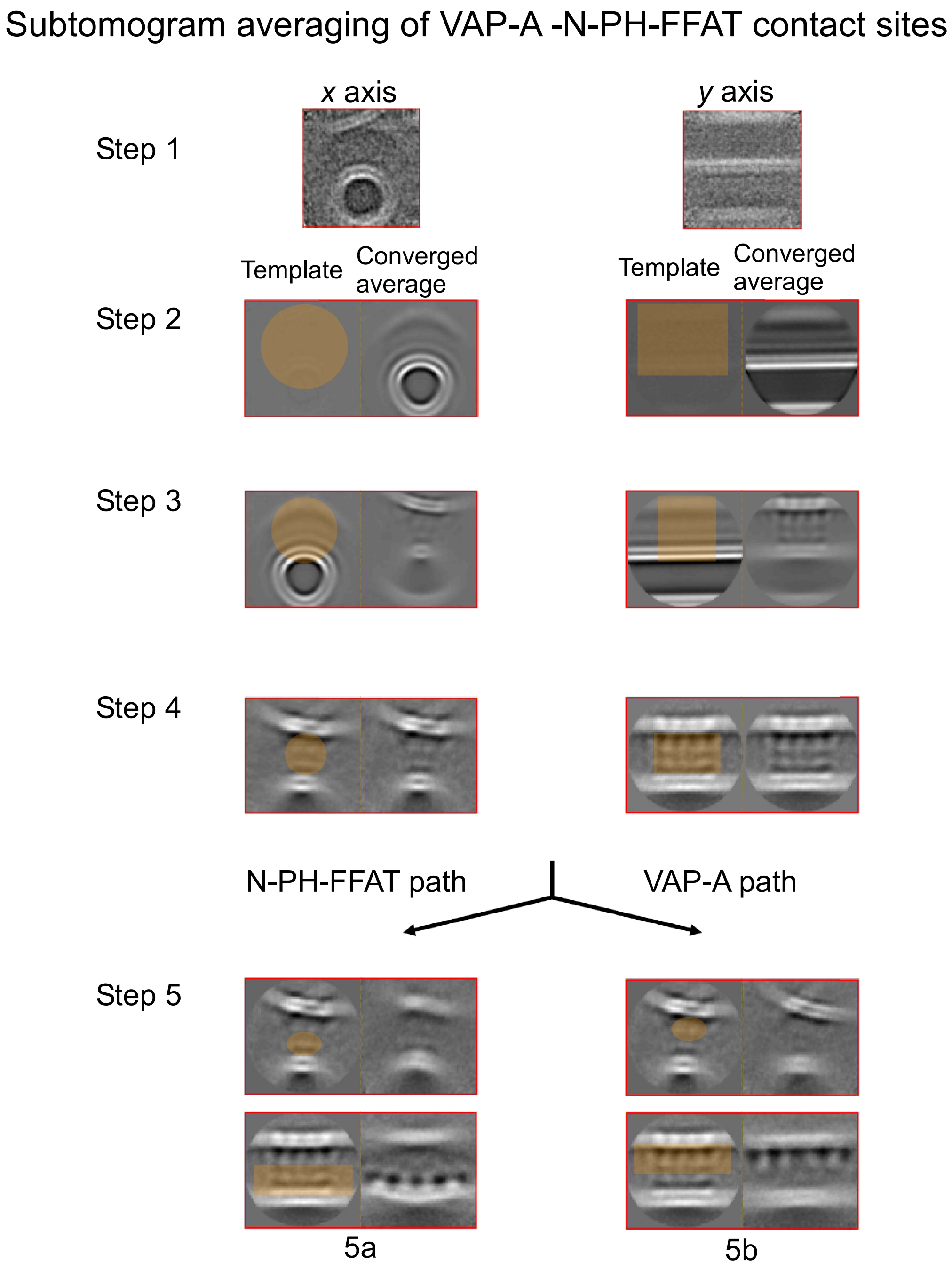

Supplementary Figure 4a

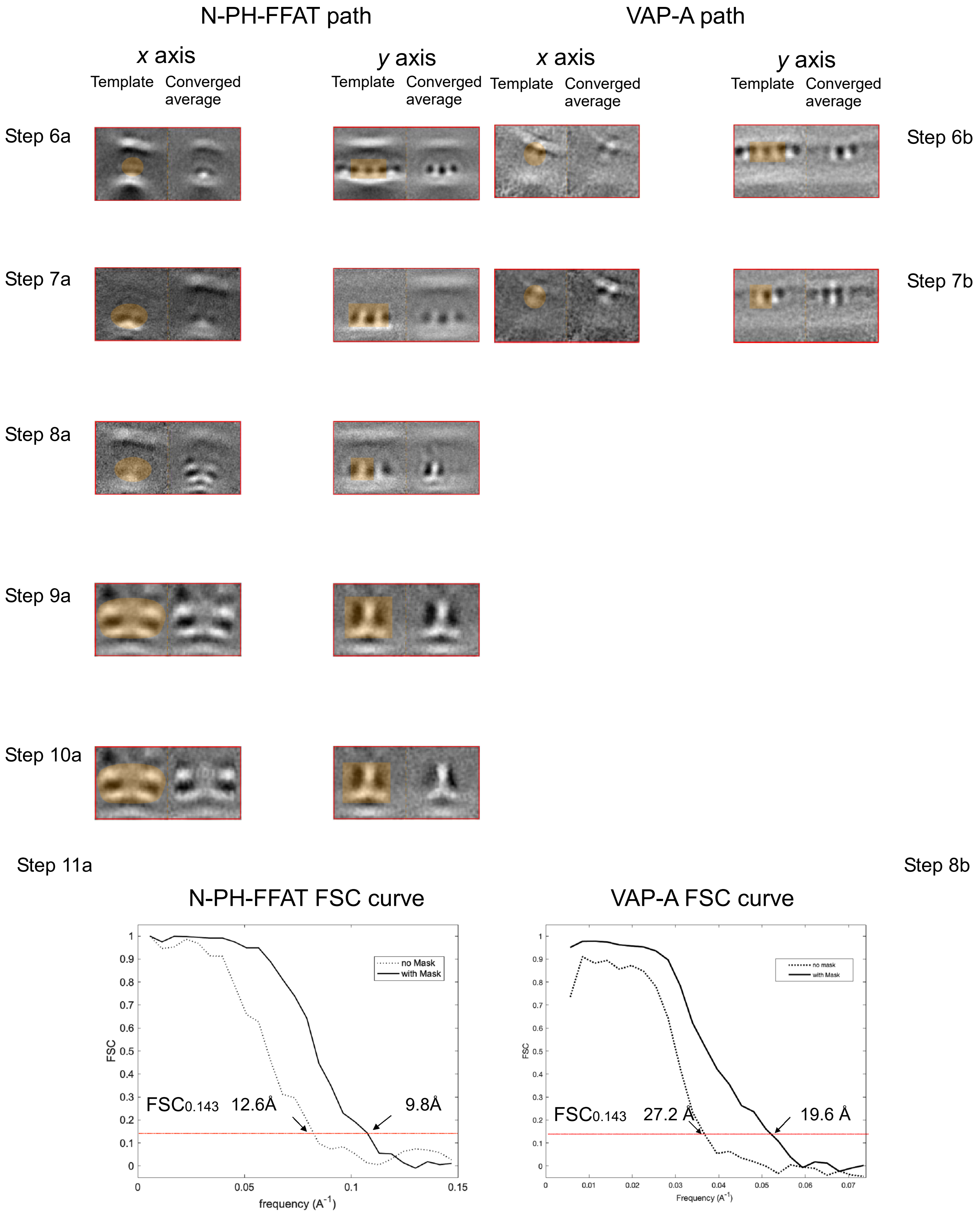

Supplementary Figure 4b

Supplementary Figure 4 Detailed pipeline of subtomogram averaging

All steps of the following pipeline were performed under Dynamo (Castaño-díez et al., 2012; Castaño-Díez et al., 2017).

Step 1 During the first step the whole dataset composed of 63,348 oversampled subvolumes of 88^3 voxels (59.8^3 Å) belonging to class III (Figure 4b) were extracted from binned 4 (voxel size = 6.8 Å) tomograms, from which an initial model-free template was obtained by averaging a set of 2,000 randomly chosen subvolumes.

Step 2 Membranes from tubes and vesicles were used during the first rounds as references to align apposed membranes. A cylindrical mask covering half of the tube and the upper region corresponding to vesicles membrane was selected. Particles were binned 2X and the angular search was restricted to a cone range of 24° (steps of 6°) and to an in-plane range of 32° (steps of 8°). Particles were allowed to shift 2 voxels along the x, y and z axes. The low-pass filter was set to 40 Å.

Step 3 Once the membranes were aligned, two lines of density corresponding to the proteins involved in the contact were observed between the apposed membranes. During step 3 we reduced the size of the cylindric mask to align the region occupied by proteins. The angular search for binned 2 particles was set to a cone range of 16° (steps of 4°) and an in-plane range of 24° (steps of 6°).

Step 4 When proteins from both sides of the membrane were observed, the size of the aligned subvolumes was reduced to 72^3 voxels (49^3 Å), being the center of box that of the contact zone, i. e. the midpoint between the external layer of tube and vesicle membranes. An alignment with restricted parameters, no angular search (zero degrees) and shifts of 1 voxel along the axis x, y and z, was performed as a control. Particle size was set to 36 (bin 2) and low-band pass to 40 Å.

Step 5 N-PH-FFAT bound to PI4P contained in tube membranes and VAP-A reconstituted in vesicles were aligned following independent pipelines. The goal of this step was to achieve a better alignment of the individual components of the contact zone.

A mask covering either N-PH-FFAT (step 5a) or VAP-A (step 5b) was chosen. Angular search was restricted to a cone range of 4° (increments of 1°) and an in-plane range of 4° (increments of 1°). Shifts were set to 8, 2 and 2 along *x*, *y* and *z*, where *x* corresponds to the tube axis. Particle size and low-band pass were kept as in last step.

Step 6 a,b As a result of the alignment described in step 5, four subunits of both N-PH-FFAT and VAP-A were clearly distinguished in the contact region of the aligned subvolumes. The goal of this step was to align the two subunits located in the central region of the box. Angular search parameters were kept to 4 ° (steps of 1°) for both cone and in-plane ranges. Shifts were also kept to 8, 2, 2, and particle separation was set to 8 voxels (5.4 Å) to decrease the number of subvolumes and select those with the best correlation coefficient (CC). Particle size and low-band pass were kept as before, i. e. 36 (bin 2) and 40 Å, respectively.

Step 7 a,b Aligned subvolumes with dimensions of 104^3 voxels (35.4^3 Å) were extracted from binned 2 (voxel size = 3.4 Å) tomograms. An alignment with restricted parameters, zero degrees for angular search and shifts of 1, 1, 1 voxel, was performed as a control to confirm that the new extraction didn't modified the result observed in step 6b. Particle size was set to 52 (bin 2) and low-band pass to 25.2 Å. The alignment mask covered the lower central region of the box.

The following steps were only performed over the N-PH-FFAT path.

Step 8a The alignment mask was restricted to one of the two subunits of N-PH-FFAT observed in the template. Angular search was set to 4° (increments of 1°) for both, cone and in-plane range. Shifts were set to 16, 2 and 2 voxels. Both membranes, that from PI4P tubes and that from VAP-A containing vesicles are still observed in the resulting average. Particle size and low-band pass were set to 36 voxels (bin 2) and 25.2 Å, respectively.

Step 9a Aligned subvolumes were *subboxed* to reduce the box size to 52^3 voxels (17.7^3 Å) and to shift the center of the box towards the aligned subunit of N-PH-FFAT. A restricted alignment was run to confirm that subvolumes were properly *subboxed*. Symmetry was set to c2 based on the evident two-

fold symmetry observed, the symmetry test performed under Dynamo and on biochemical data that have shown that N-PH-FFAT is a dimer (Jamecna *et al*. 2019).

Step 10a The last step consisted of an adaptive band-pass filter alignment (FSC set to 0.143). Initial parameters of the angular search were set to a cone range of 16° (increments of 4°) and in-plane range of 180° (increments of 10°) while shifts were set 8, 6, 6 voxels along the *x*, *y* and *z* axes. Symmetry was kept as c2. Both, the angular search and shifts were gradually decreased, and the band-pass filter moved towards higher resolution.

Step 11a, 8b The final converged average consisted on 15,597 and 6,948 subvolumes for N-PH-FFAT and VAP-A respectively, and their respective FSC were calculated over the two half-averages obtained upon randomly splitting the subvolumes in two datasets.

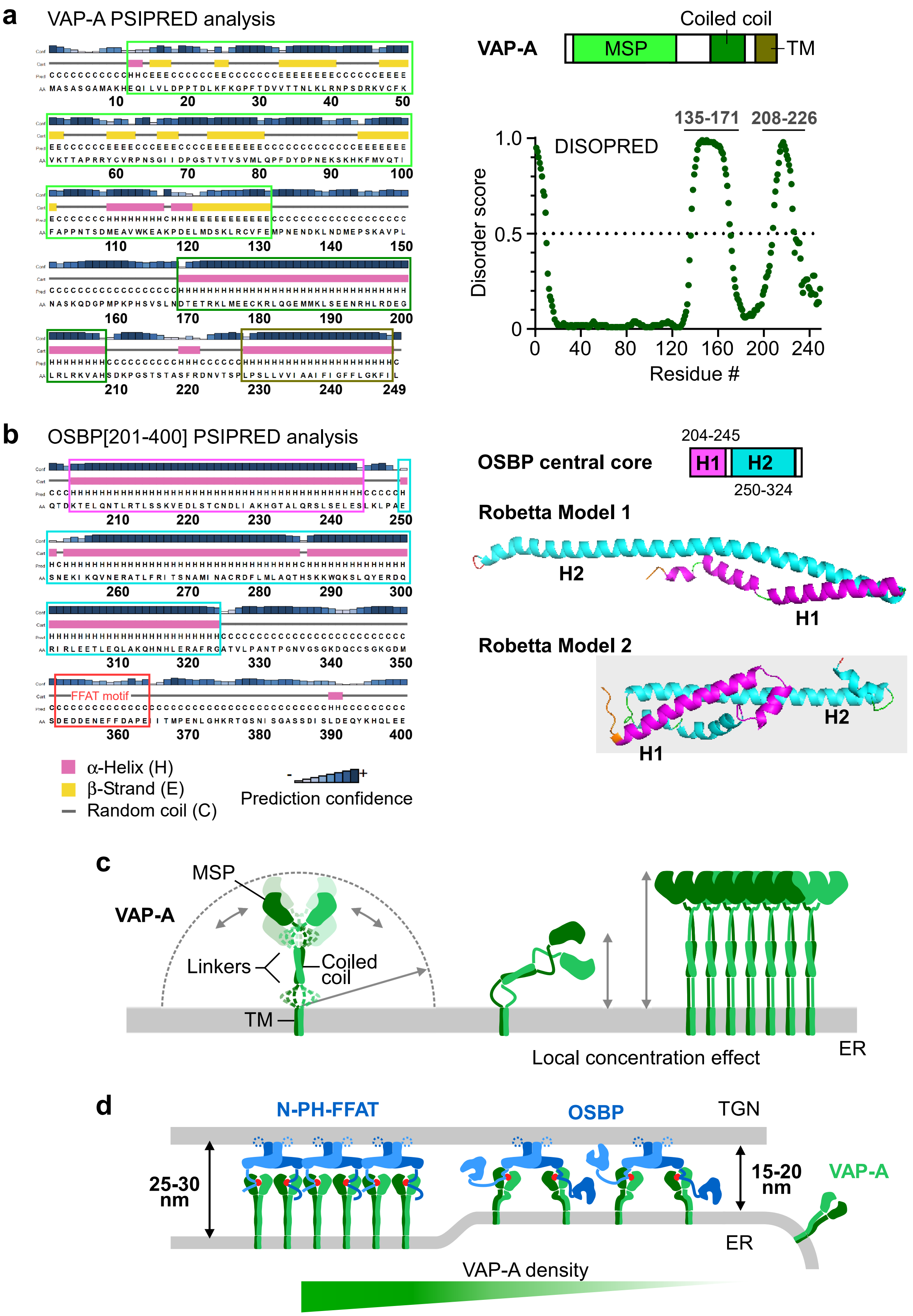
a
VAP-A PSIPRED analysis
VAP-A
MSP
Coiled coil
TM
135-171
208-226
1.0
0.5
0
DISOPRED
Disorder score
0 40 80 120 160 200 240
Residue #
b
OSBP[201-400] PSIPRED analysis
FFAT motif
OSBP central core
204-245
H1
H2
250-324
Robetta Model 1
Robetta Model 2
α-Helix (H)
β-Strand (E)
Random coil (C)
Prediction confidence
c
MSP
VAP-A
Linkers
Coiled coil
TM
Local concentration effect
ER
d
N-PH-FFAT
OSBP
TGN
25-30 nm
15-20 nm
VAP-A
ER
VAP-A density

Supplementary Figure 5. Prediction and model of VAP-A and OSBP

(a) Secondary structure prediction of human VAP-A by PSIPred. VAP-A is made of the MSP domain (light-green square), a putative CC region (green square), a transmembrane helix (brown square) and two unstructured linkers SL1 (aa135-171) and SL2 (aa208-226) that flank the CC region. Disordered regions predicted by Disopred program.

(b) Secondary structure prediction of human central core OSBP (aa201-400) by PSIPred program. OSBP (aa201-400) is helix H1 (aa204-245) and helix H2 (aa249-325) and a disordered region 326-408 that contains the FFAT motif. Models of central core OSBP computed by Robetta.

(c) Schematic representation of VAP-A organization at the ER membrane. The disordered linkers of VAP-A allow VAP-A to explore a large space to bind its interactors. When concentrated in a cluster VAP-A can extend to 17 nm from ER membrane.

(d) Organization of VAP-A in ER Golgi MCS depending on its local concentration. VAP-A concentrate in flat membrane region of MCS and adapt its concentration to its interactors. Fewer molecules of VAP-A are engaged in MCS containing OSBP with bulky ORD domains than with N-PH-FFAT. This results in shorter intermembrane distances in MCSs containing OSBP than N-PH-FFAT.

REFERENCES

1. Jamecna, D. *et al.* An Intrinsically Disordered Region in OSBP Acts as an Entropic Barrier to Control Protein Dynamics and Orientation at Membrane Contact Sites. *Dev. Cell* **49**, 220–234 (2019).

2. de Saint-Jean, M. *et al.* Osh4p exchanges sterols for phosphatidylinositol 4-phosphate between lipid bilayers. *J. Cell Biol.* **195**, 965–978 (2011).

3. Zheng, S. Q. *et al.* MotionCor2: Anisotropic correction of beam-induced motion for improved cryo-electron microscopy. *Nat. Methods* **14**, 331–332 (2017).

4. Schneider, C. A., Rasband, W. S. & Eliceiri, K. W. NIH Image to ImageJ: 25 years of image analysis. *Nat. Methods* **9**, 671–675 (2012).

5. Mastronarde, D. N. Automated electron microscope tomography using robust prediction of specimen movements. *J. Struct. Biol.* **152**, 36–51 (2005).

6. Xiong, Q., Morphew, M. K., Schwartz, C. L., Hoenger, A. H. & Mastronarde, D. N. CTF determination and correction for low dose tomographic tilt series. *J. Struct. Biol.* **168**, 378–387 (2009).

7. Castaño-Díez, D., Kudryashev, M., Arheit, M. & Stahlberg, H. Dynamo Catalogue: Geometrical tools and data management for particle picking in subtomogram averaging of cryo-electron tomograms. *J. Struct. Biol.* **178**, 135–144 (2017).

8. Castaño-díez, D., Kudryashev, M., Arheit, M. & Stahlberg, H. Dynamo : A flexible , user-friendly development tool for subtomogram averaging of cryo-EM data in high-performance computing environments. *J. Struct. Biol.* **178**, 139–151 (2012).

9. Mastronarde, D. N. & Held, S. R. Automated tilt series alignment and tomographic reconstruction in IMOD. *J. Struct. Biol.* **197**, 102–113 (2017).

10. Goddard, T. D., Huang, C. C. & Ferrin, T. E. Visualizing density maps with UCSF Chimera. *J. Struct. Biol.* **157**, 281–287 (2007).

11. Raman, S. *et al.* Structure prediction for CASP8 with all-atom refinement using Rosetta. *Proteins Struct. Funct. Bioinforma.* **77**, 89–99 (2009).

12. Song, Y. *et al.* High-resolution comparative modeling with RosettaCM. *Structure* **21**, 1735–1742 (2013).

13. Yang, J. *et al.* Improved protein structure prediction using predicted interresidue orientations. *Proc. Natl. Acad. Sci. U. S. A.* **117**, 1496–1503 (2020).

14. Kelley, L. A., Mezulis, S., Yates, C. M., Wass, M. N. & Sternberg, M. J. E. The Phyre2 web portal for protein modeling, prediction and analysis. *Nat. Protoc.* **10**, 845–858 (2016).

15. Buchan, D. W. A. & Jones, D. T. The PSIPRED Protein Analysis Workbench: 20 years on. *Nucleic Acids Res.* **47**, W402–W407 (2019).

16. Schrödinger, L. The PyMOL Molecular Graphics System, Version 1.3r1. (2010).

17. Abraham, M.J. *et al.* GROMACS: High performance molecular simulations through multi-level parallelism from laptops to supercomputers. *SoftwareX* **1**–**2**, 19–25 (2015).

18. Duchesne, L., Gentili, D., Comes-Franchini, M. & Fernig, D. G. Robust ligand shells for biological applications of gold nanoparticles. *Langmuir* **24**, 13572–13580 (2008).
