# Supplementary material for: Nanoscale architecture of a VAP-A-OSBP tethering complex at membrane contact sites
Source: Nat Commun. 2021 Jun 8;12:3459. doi: 10.1038/s41467-021-23799-1 (PMC8187361; doi:10.1038/s41467-021-23799-1)
Supplement: Supplementary file 3 — Description of Additional Supplementary Files [file 41467_2021_23799_MOESM3_ESM.docx]

**Description of Additional Supplementary Files**

File Name: **Supplementary Movie 1**

Description: **Cryo-tomogram of VAP-A//N-PH-FFAT Membrane Contact Sites**

File Name: **Supplementary Movie 2**

Description: **Cryo-tomogram of VAP-A in ribbons//N-PH-FFAT Membrane Contact Sites**

File Name: **Supplementary Movie 3**

Description: **Cryo-tomogram of VAP-A//OSBP Membrane Contact Sites**

File Name: **Supplementary Movie 4**

Description: **Cryo-tomogram of VAP-A//N-PH-FFAT Membrane Contact Sites with tongue**
